# Short Is Beautiful: Dimensionality and Measurement Invariance in Two Length of the Basic Psychological Need Satisfaction at Work Scale

**DOI:** 10.3389/fpsyg.2018.00965

**Published:** 2018-06-14

**Authors:** Mårten Eriksson, Eva Boman

**Affiliations:** Department of Social Work and Psychology, Faculty of Health and Occupational Studies, Gävle University, Gävle, Sweden

**Keywords:** confirmatory factor analysis, measurement invariance, basic need satisfaction scale, self-determination theory, motivation

## Abstract

Self-determination theory proposes that all humans have three intrinsic psychological needs: the needs for *Autonomy*, *Competence*, and *Relatedness*. These needs take different forms in different areas of life. The present study examines the psychometric properties of a Swedish version of the Basic Psychological Need Satisfaction at Work (BPNS-W) scale. The fit of 10-factor structures previously suggested for related versions of the scale were compared. Cross-sectional data from 1,200 participants were examined in a confirmatory factor analysis framework. Both the original 21-item version and a reduced 12-item version of the BPNS-W were examined. The General Health Questionnaire was used for validation. The results supported a three-factor solution with correlated error variances for the reversed items. Invariance testing of the long and short scales gave best support to the short scale, for which partial scalar invariance was achieved. The external validity of the short scale was supported by a hierarchical regression analysis in which each need made a unique contribution in predicting psychological well-being. In conclusion, the results corroborate a three-factor structure of BPNS-W. Although not perfect the short scale should, it is argued, be preferred over the long version. Directions for the future development of the scale are discussed.

## Introduction

Self-determination theory (SDT; e.g., Deci and Ryan, 2000; Ryan and Deci, 2000) is a theory of human motivation, psychological growth, and well-being. In SDT, three basic psychological needs are proposed: *autonomy*, *competence*, and *relatedness*. The satisfaction of these basic needs is referred to as BNS [Basic Need Satisfaction or Basic Psychological Need Satisfaction (BPNS)]; the literature is inconsistent on whether or not to include P for “psychological”). The three needs are viewed as being innate in the same way as physiological needs are (e.g., the need for food and drink). However, in contrast to physiological needs and needs in psychological theories such as that of Maslow (1943), the needs in SDT are not reduced when satisfied. Instead, need satisfaction in SDT motivates individuals to perform subsequent need-fulfilling behaviors (Deci and Ryan, 2000; van den Broeck et al., 2010). *Autonomy* reflects the extent to which an individual experiences her behavior as self-initiated and in accordance with *her integrated sense of self* (Ryan and Deci, 2000). *Competence* has its roots in children’s propensity to explore the world. It later develops into the need to have an effect on, or master, the environment in some way, and to gain the appreciation that comes with such behavior (e.g., positive feedback; Deci and Ryan, 2000). Finally, *Relatedness* concerns the need to experience love and care as well as to express love and care for others (Deci and Ryan, 2000). Each need is supposed to predict, independently, various outcomes such as motivation, psychological health, psychological growth, and well-being (Deci and Ryan, 2000; Ryan and Deci, 2000).

The present study concerns the measurement of BPNS at work (BPNS-W). In a work setting, the need for *Autonomy* is satisfied when a worker experiences a sense of freedom and choice when doing the job (Deci and Ryan, 2000). Hence, it is the subjective experience of *Autonomy* that is crucial in BPNS, and it should not be confused with related concepts in theories of organizational psychology in which *Autonomy* and control refer to task characteristics (van den Broeck et al., 2010). The need for *Competence* at work is satisfied when a worker is engaged in challenging tasks, allowed to apply and extend her skills, and appreciated for the effort and therefore experiences effectiveness at work (Deci and Ryan, 2000; Brien et al., 2012). The need for *Relatedness* at work is satisfied when a worker establishes mutually caring bonds with colleagues at work (Deci and Ryan, 2000; Brien et al., 2012). This need is similar to other concepts in organizational psychology, such as social support (van den Broeck et al., 2010).

A prerequisite for evaluating the precise role of the three basic needs in SDT, as well as for further developing the theory, is to find ways of assessing *Autonomy*, *Competence*, and *Relatedness* properly. Initially, instruments assessing *Autonomy*, *Competence*, and *Relatedness* were typically used as components in rather complex models demonstrating how various social determinants affect the satisfaction of the three needs, a satisfaction found to be crucial for intrinsic motivation as well as psychological growth and well-being (Deci and Ryan, 2000; Ryan and Deci, 2000). Subsequently, scales have been developed to assess the three needs both at a general level (e.g., Gagné, 2003; Johnston and Finney, 2010; Sheldon and Hilpert, 2012) and in specific domains such as education (Filak and Sheldon, 2003; Longo et al., 2016) and e-learning (Roca and Gagné, 2008; Sørebø et al., 2009), interpersonal relationships (La Guardia et al., 2000), sports (Vlachopoulos and Michailidou, 2006; Adie et al., 2008; Vlachopoulos, 2008; Vlachopoulos et al., 2010; Ng et al., 2011; Sheldon and Hilpert, 2012) and at work (Deci et al., 2001; Arshadi, 2010; van den Broeck et al., 2010; Brien et al., 2012; see van den Broeck et al., 2016, for a review). The various instruments are similar in many respects but include different numbers of items and have been adapted for different settings and applied to different populations. Measures of BPNS have continued to receive support from other domains. For example, BPNS has been found to be positively related to prosocial behavior (Gagné, 2003), attachment security (La Guardia et al., 2000), motivation (Sørebø et al., 2009; Arshadi, 2010), and subjective vitality in sports (Adie et al., 2008).

A growing interest in the psychometric properties of the scales can be seen in publications from the last decade, and both some strengths as well as several limitations have been identified. In particular, concern has been raised about the balance between the three needs, the issue of negative need fulfillment and which factor structure is the most appropriate. Several new versions of the scale have been developed in response to these concerns.

In the original, 21-item BPNS-W scale (Deci et al., 2001), seven items assess *Autonomy*, six assess *Competence* and eight assess *Relatedness*. This imbalance has been questioned, and the use of an equal number of items assessing each need advocated (Sheldon and Hilpert, 2012).

The nature of the low endpoint of BPNS scales has also been discussed. It has been argued (Sheldon and Hilpert, 2012; Chen et al., 2015; Longo et al., 2016) that need frustration is not simply the reverse of need fulfillment but a separate though related construct that deserves to be investigated in its own right. In particular, need frustration is claimed to motivate actions that promote the fulfillment of the need in question in a way that neither high nor low values on need fulfillment does (Sheldon and Gunz, 2009). Different attempts have therefore been made to develop scales that assess need fulfillment and need frustration separately and not simply as the opposites of each other. Such scales have been proposed by Sheldon and Hilpert (2012; the Balanced Measure of Psychological Need Scale, BMPNS, domain general, comprising 18 items), Chen et al. (2015; the Basic Psychological Need Satisfaction and Frustration Scale for the work domain, BPNSFS, comprising 24 items), and Longo et al. (2016; the need satisfaction and frustration scale, NSFS, designed for educational and work contexts, comprising 18 items).

There is also an ongoing discussion on the factor structure of measures of the satisfaction (and frustration) of the three psychological needs. For example, Deci et al. (2001) claimed that the BPNS scales measure the needs of *Autonomy*, *Competence*, and *Relatedness* both separately and as a composite measure of general need satisfaction. The idea of adding different subscales together to achieve a grand total is not unique to measures of basic needs but may cause problems because each subscale is expected to contribute uniquely to a given outcome and at the same time be interchangeable in a grand total. In dimensional terms, it is a question of whether psychological needs are best represented by one latent factor model or a three-factor model.

Moreover, the original BPNS scales have followed the common psychometric tradition (e.g., Furr, 2011) of including both positively and negatively worded items. The reasons for including negatively worded items are usually to reduce extreme response bias, introduce more variation among the items, and guard against acquiescent bias (Spector, 1992). This mix of positively and negatively worded items is assumed not to affect the factor structure. However, research from personality measurement (Horan et al., 2003; DiStefano and Motl, 2006) have suggested that answers to negatively worded questions disclose individual differences in terms of response styles, and such items are therefore best represented in a factor structure that includes a special method factor for the negatively worded items.

**FIGURE 1:**
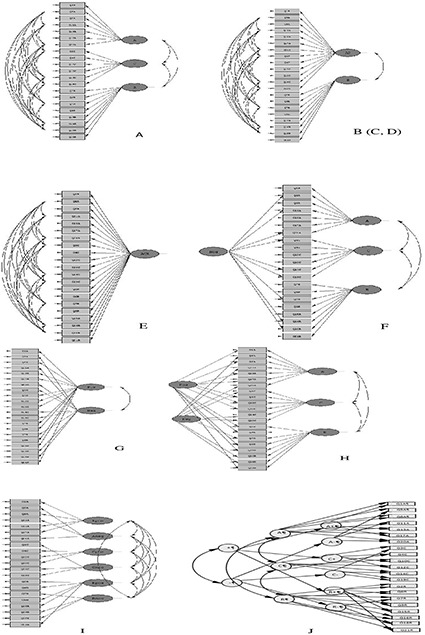
Suggested factor structures **(A–J)** to scales measuring basic psychological need satisfaction.

We have identified 10 different factor structures that have previously been suggested for scales relating to BPNS (**Figure 1**). Model A is the basic three-factor structure with correlated needs suggested by SDT (e.g., Deci et al., 2001) and previously supported by Brien et al. (2012) using a 12-item French version of the Basic Psychological Needs at Work Scale (BPNWS), Ng et al. (2011) using a 15-item need scale for sports, van den Broeck et al. (2010) using an 18-item Dutch scale called the Work-related Basic Need Satisfaction Scale (WBNS), and Vlachopoulos and Michailidou (2006) using a 12-item basic need scale for sport in Greece. In addition to model A, Vlachopoulos and Michailidou (2006) investigated the fit of four other models (Models B–E), all of which were outperformed by Model A. In Model B, indicators of *Autonomy* and *Competence* were merged into a single factor and indicators of *Relatedness* retained as a second factor. In Model C, indicators of *Autonomy* and *Relatedness* were merged into a single factor and *Competence* retained as a second factor. In Model D, indicators of *Relatedness* and *Competence* formed one factor and indicators of *Autonomy* formed another. In Model E, all indicators loaded directly onto a single (basic need) factor (In contrast to the studies of Vlachopoulos and Michailidou, the error variance for the reversed items in Models A–E are allowed to correlate, as in **Figure 1**—see below for a discussion on this point. Models C and D have a similar structure to Model B and are therefore not depicted separately in **Figure 1**).

Johnston and Finney (2010) found weak support for Model A for the general basic need satisfaction scale comprising 21 items. In particular, they reported that some items had high error variance or loaded onto more than one need. However, the fit improved considerably when five poorly worded items were excluded (of which four concerned *Autonomy* and one *Relatedness*), and the remaining negatively worded items were grouped into a fourth, method-effect factor. A 21-item version of this structure corresponds to Model F in **Figure 1**. Model G is a two-factor valence model tested by both Longo et al. (2016) and Sheldon and Hilpert (2012) in which negative and positive items loaded on different factors. This model was not supported by either study. Model H received the best support in Sheldon and Hilpert’s (2012) study and consisted of one factor for each need (correlated) and two uncorrelated method factors reflecting satisfaction and dissatisfaction, respectively, in a multi-trait multi-method design (MTMM-CTUM). Model I is a six-factor model with both a satisfaction factor and a frustration factor attached to each need. This model was found to be superior to Model A by Chen et al. (2015) using the BPNSFS. The model best supported in the study by Longo et al. (2016) consisted of six first-order latent factors (each need divided into a positive and a negative factor), three higher order factors corresponding to each need, and one positive and one negative higher order valence factor as depicted in Model J.

### The Present Study

As discussed above, several shortcomings in scales measuring basic needs have been identified, and many alternative versions have been suggested to remedy at least some of the problems. However, it is unclear to what extent the criticism applies to scales in all domains and populations. The dimensionality of the original 21-item version of BPNS-W scale has only been tested explicitly using confirmatory factor analysis (CFA), by Longo et al. (2016) in a study comprising 243 participants. The need for more studies to be conducted is recognized. Considering the large number of studies that have employed the BPNS-W scales, and their relative success (at least in complex models), further CFA studies on the dimensionality of BPNS-W scale with higher numbers of participants are needed.

The present study concerns the psychometric properties of a Swedish version of the BPNS-W scale. We investigated the fit of the 10 previously discussed factor structures of BPNS scales using CFA. The three basic needs, of *Autonomy*, *Competence*, and *Relatedness*, are central in Models A, F, H, I, and J, although they may be split into positive and negative dimensions or moderated by method factors. In contrast, Models B–E and Model G do not include the three needs as separate factors. Better support for these latter models over the former would indicate imperfections in the instrument.

In Models F and H, negative items were allocated to particular method factors. We replicated these models by grouping the reversed items in a specific method factor. This was compared with models in which the error variances of the reversed items were allowed to correlate covariate (see, for example, Brown, 2015). The latter procedure is similar to a correlated trait—correlated uniqueness model (CTCU) as described by Marsh and Grayson (1995), although there should be more than one method factor in a true CTCU model.

We also investigated whether a shortening of the scale can solve problems of high-error variance or cross-loadings on non-intended factors, as found by Johnston and Finney (2010). To this end, indicators with reasonable error variance and loadings on designated factors were retained, and indicators loading high on more than one factor avoided. We aimed for a balance between the numbers of indicators for each factor, including both positively and reversed wording to reduce bias associated with acquiescence or affirmation. At least four indicators for each subscale were to be retained, as fewer indicators per factor is a common source of inadmissible solutions, particularly for models including method factors (cf. Brown, 2015). However, a reduction in the number of items on a scale is often associated with limited coverage of the target concept and lower internal consistency. Hence, any reduction must be done with care.

Furthermore, we investigated the measurement invariance of both the full 21-item scale and a reduced scale across two random samples. Measurement invariance is important because scalar invariance (equal number of factors, equal loadings, and equal intercepts in the two samples) at least is considered necessary for calculations of tests based on mean differences of manifest scores (e.g., Cheung and Rensvold, 2002; van de Schoot et al., 2012; Brown, 2015). Measurement invariance across random samples tests basic psychometric assumptions of the construct. If affirmative support for invariance is found, follow-up studies between specific groups are needed before studies of mean differences can be performed in applied research. If no support for invariance between random samples can be found, future research should focus instead on finding better indicators. Hitherto, it has only been tested for a few times with BPN scales. Vlachopoulos and Michailidou (2006) reported partial scalar invariance across two random samples of the BPNES. Brien et al. tested the measurement invariance of a French version of the BPNWS between a French sample and a Canadian sample, and again, only partial scalar invariance was supported.

Finally, the validity of the Swedish versions was investigated in relation to a broad measure of psychological well-being. Psychological well-being is one of several applicable standards relevant to the validity of BPNS-W.

The following research questions were formulated:

1. Which of the 10 factor structures discussed above best fit data generated by the Swedish version of the BPNS-W scale?
2. Can the Swedish version of the BPNS-W scale be improved simply by eliminating some items?
3. Is the Swedish version of BNS-W (original and reduced) invariant across two random samples?
4. Is each need (*Autonomy*, *Competence*, and *Relatedness*) uniquely related to psychological well-being in the BPNS-W?

## Materials and Methods

### Participants and Procedure

Data from 1,200 respondents were collected by students at the University of Gävle as part of a course assignment. Participation was voluntary, and consent given after information on the project was presented (informed consent). All data were collected in accordance with the ethical guidelines of the Helsinki Declaration, the American Psychological Association (APA), and the Swedish Research Council. In all, 0.055% of the data from the 21 variables measuring basic needs was missing. These data were substituted with the mean value of respondents of the same gender and occupation on the missing variable.

The data were randomly divided into two equal parts. The first sample of 600 participants (calibration sample, CS) was used to explore the factor structure and reduction of the scale length. The second sample of 600 participants (verification sample, VerS) was used to verify that the psychometric properties found for the reduced scale could be replicated in another sample from the same population. CS and VerS were also used in the analyses of measurement invariance. A third subsample, of 419 participants (validation sample, ValS), consisted of individuals who had provided data on their psychological well-being. These were used for external validation.

Calibration sample consisted of 384 women and 207 men (plus nine participants who did not disclose their gender). Around half (289 participants) were below 45 years of age, 284 were older, and 27 did not disclose their age. Participants worked as civil servants (342), nurses (196), teachers (41), or in industry (21).

VerS consisted of 399 women and 193 men (plus eight participants who did not disclose their gender). A good half (305 individuals) were below 45 years of age, 260 were older, and 35 did not disclose their age. Again, participants worked as civil servants (321), nurses (212), teachers (43), or in industry (24).

ValS consisted of 419 participants, of whom 253 were women and 165 men. One participant preferred not to provide information on gender. Around half (228 participants) were below 45 years of age, 189 were older, and 2 participants did not disclose information on their age. Participants in this group worked as civil servants (234), nurses (140), or in industry (45).

### Measures

All participants completed the Swedish version of the BPNS-W scale. The form was translated into Swedish by the first author (**Table 1**) and then back-translated into English by a bilingual, native-English-speaking researcher, after which differences between the two English versions were discussed (cf. Brislin et al., 1973). Only minor differences in style between the back-translated and the original version were found. The original form was retrieved from the SDT website^1^. The scale contained 21 statements divided into seven items measuring *Autonomy*, six measuring *Competence*, and eight items measuring *Relatedness*. The wording of three items for each need was reversed. Responses were recorded on a 7-point scale ranging from “not at all true” (1) to “very true” (7).

**Table 1:** Multivariate normality of the BPNS items (*N* = 1,200).

	Means		*SD*		Skewness		Kurtosis
***Autonomy* (composite reliability: 7 items = 0.63; 4 items = 0.63)**		
Q1^∗^. Jag har goda möjligheter att påverka hur mitt arbete ska utföras	5.13		1.25		-0.71		0.43
(I feel like I can make a lot of input into deciding how my job gets done)							
Q5. Jag känner mig pressad på arbetet	4.13		1.64		-0.03		-0.85
(I feel pressured at work) (R)							
Q8.^∗^ Jag kan fritt uttrycka vad jag tycker och tänker på jobbet	5.19		1.49		-0.84		0.22
(I am free to express my ideas and opinions on the job)							
Q11. När jag är på jobbet måste jag göra vad jag blir tillsagd	3.58		1.66		0.26		-0.74
(When I am at work, I have to do what I am told) (R)							
Q13^∗^. Mina känslor beaktas på jobbet	4.76		1.42		-0.56		-0.08
(My feelings are taken into consideration at work)							
Q17. Jag känner att jag kan vara mig själv på jobbet	5.73		1.25		-1.36		2.24
(I feel like I can pretty much be myself at work)							
Q20^∗^. Jag har få påverkansmöjligheter i mitt arbete	4.64		1.69		-0.37		-0.82
(There is not much opportunity for me to decide for myself how to go about my work) (R)							
***Competence* (composite reliability: 6 items = 0.53; 4 items = 0.60)**		
Q3. Jag behärskar inte riktigt mitt jobb	5.60		1.81		-1.40		0.78
(I do not feel very competent when I am at work) (R)							
Q4.^∗^ På jobbet får jag uppskattning för mitt arbete	4.91		1.40		-0.51		-0.17
(People at work tell me I am good at what I do)							
Q10.^∗^ Jag har lärt mig nya intressanta färdigheter på jobbet	5.53		1.31		-0.96		0.66
(I have been able to learn interesting new skills on my job)							
Q12.^∗^ De flesta dagar känner jag att jag uträttat något på jobbet	5.77		1.20		-1.34		2.32
(Most days I feel a sense of accomplishment from working)							
Q14.^∗^ Jag har inte stora möjligheter att visa vad jag går för på jobbet	4.99		1.69		-0.62		-0.63
(On my job I do not get much of a chance to show how capable I am) (R)							
Q19. Jag känner ofta jag saknar kompetens i mitt arbete	5.69		1.38		-1.17		0.78
(When I am working I often do not feel very capable) (R)							
***Relatedness* (composite reliability: 8 items = 0.78; 4 items = 0.63)**		
Q2^∗^. Jag gillar mina arbetskamrater	6.11		1.00		-1.23		1.54
(I really like the people I work with)							
Q6. Jag kommer överens med mina arbetskamrater	6.08		0.97		-1.52		3.54
(I get along with people at work)							
Q7 Jag håller mig för mig själv på jobbet	5.81		1.46		-1.24		0.70
(I pretty much keep to myself at work) (R)							
Q9. Jag betraktar mina arbetskamrater som mina vänner	5.08		1.45		-0.56		-0.25
(I consider the people I work with to be my friends)							
Q15^∗^. Mina arbetskamrater bryr sig om mig	5.65		1.12		-0.72		0.24
(People at work care about me)							
Q16^∗^. Jag har inte många nära vänner på jobbet	4.63		1.84		-0.35		-0.99
(There are not many people at work that I am close to) (R)						
Q18. Mina arbetskamrater gillar mig inte	6.26		0.97		-1.81		3.92
(The people I work with do not seem to like me much) (R)						
Q21^∗^. Människor är vänliga på mitt jobb	5.87		1.10		-1.26		1.90
(People at work are pretty friendly toward me)							

*Asterisk “^∗^” denotes items maintained in the short version; “(R)” denotes reversed items*.

Psychological well-being was measured by the short version of Goldberg’s (1972) General Health Questionnaire. We used the Swedish version described by Sconfienza (1998), which contains 12 items and for which responses are given on a 4-point scale ranging from “disagree” (1) to “agree completely” (4). Sconfienza reports an internal consistency of 0.80–0.84 for this version of GHQ. Data from the present study lay within that range (0.81).

### Plan of Analysis

The study involved analyzing the fit of 10 models, 2 scale lengths, and 3 subsamples. A guide to the order of analyses might, therefore, be needed, as follows. The total sample of 1,200 participants was used in the initial screening for normality used to determine the appropriate estimator function. The fit of the 10 pre-specified factor structures was first evaluated on CS using the 21-item scale. The factor loadings from the best models were then used to develop a shorter, 12-item version using CS first, and then using VerS to verify it. Factorial invariance was investigated for both the 21-item and 12-item scales of Model A using multi-group (CS/VerS) CFA. Finally, ValS was used to validate the 12-item scale using multiple regression analysis.

### Statistical Analyses

Because the research questions concern the fit of a set of pre-specified factor models, covariance between error terms and measurement invariance evaluation, CFA was the principal method of analysis. These analyses were conducted in LISREL 8.80.

As indices of model fit, root mean square error of approximation (RMSEA), standardized root mean square residual (SRMR), comparative fit index (CFI), and Tucker–Lewis index (TLI) are reported as being recommended by Hu and Bentler (1999). Values below or “close to” 0.06 for RMSEA and below or “close to” 0.08 for SRMR are indications of a “reasonably good fit,” as are values “close to” or above 0.95 for CFI and TLI. An evaluation of all four indices is advocated, rather than using them as strict cut-off values (cf. Vandenberg and Lance, 2000). A chi-square was recorded for each examined model, which allows for comparison between nested models. Because the chi-square is dependent on the degrees of freedom (DF), chi-square over DF is also reported. The Akaike Information Criterion (AIC) was also reported on and used for comparing both nested and non-nested models. The model with lowest AIC is considered superior (Brown, 2015).

A hierarchical regression analysis was employed to investigate whether each psychological need made a unique contribution in predicting psychological well-being. This was computed in SPSS 22.0. In all analyses, a two-tailed significance level <0.05 was regarded as statistically significant.

## Results

### Data Screening

Indicator data for the whole sample were considered at approximate interval level and screened for multivariate normality. Skewness was within the -2 and +2 range for all indicators and therefore within standard criteria for normality (e.g., Field, 2009). Kurtosis was somewhat higher for a few indicators, in particular for Q6 and Q18, albeit remaining below 4. See **Table 1** for descriptive statistics. The subsequent CFA was, therefore, computed with maximum likelihood estimations, ML (See Curran et al., 1996, for a discussion on deviation from normality in CFA, sample size, choice of estimator function and effects on test power). The internal reliability of *Autonomy* (0.63) and *Competence* (0.53) were low, but adequate for *Relatedness* (0.78).

### Dimensionality

**Table 2** shows the fit indices for the seven models that converged to an admissible solution. Models A–E were computed both with uncorrelated and correlated error variance for the nine reversed items. The first two lines in **Table 2** show the chi-square and DF for Models A–E with uncorrelated error terms, followed by chi-square, DF, RMSEA, TLI, CFI, SRMR, and the AIC value for the more interesting models with a correlated error variance for the reversed items. The latter procedure resulted in a significantly better fit for all five models as determined by a chi-square test (The smallest gain in chi-square was for Model B: Dχ^2^ = 214.57, *DF* = 36, *p* < 0.01; RMSEA for the uncorrelated Model B was 0.068, TLI = 0.92, CFI = 0.93, and SRMR = 0.060).

**Table 2:** Summary of goodness of fit indices for the seven models that converged to a solution (21 items, calibration sample, *N* = 600).

	3-Factor (A; C; R) A	2-Factor (AC; R) B	2-Factor (AR; C) C	2-Factor (A; CR) D	1-Factor (ACR) E	4-Factor (A; C; R; Neg) F	2-Valence (Pos; Neg) G
Chi-square	702.85	709.70	1021.65	983.64	1130.20		
DF	186	188	188	188	189		
Chi-square/DF	3.78	3.78	5.43	5.23	5.98		
Chi-square	489.56	495.13	761.80	723.34	848.37	549.70	1060.36
DF	150	152	152	152	153	177	188
Chi-square/DF	3.26	3.26	5.01	4.76	5.54	3.11	5.64
RMSEA	0.061	0.061	0.082	0.079	0.087	0.059	0.088
90% conf	0.055–0.068	0.055–0.067	0.076–0.088	0.073–0.085	0.081–0.093	0.054–0.065	0.083–0.093
TLI	0.93	0.93	0.89	0.089	0.88	0.93	0.89
CFI	0.95	0.95	0.92	0.092	0.91	0.94	0.90
SRMR	0.049	0.049	0.060	0.059	0.060	0.051	0.068
AIC	651.56	1083.13	919.80	881.34	1004.37	657.70	1146.36

Only Models A and B with correlated errors for the reversed items, and Model F, met the criteria for good model fit recommended by Hu and Bentler (1999). The fit of Models A and B was almost identical, although the AIC value was lower for Model A. Model F had a lower RMSEA, of 0.059 compared with the 0.061 of Models A and B. Yet, the SRMR values were slightly lower for Models A and B (0.049) compared with that for Model F (0.051). In addition, CFI was higher (0.95) for Models A and B compared with 0.94 for Model F. Thus, the differences in fit between Models A, B, and F are marginal and go in both directions. It seems therefore fair to consider them as equally good. The fit of Models C–E and G depart from the recommended values on two to three fit indices. Moreover, as can be seen from **Table 2**, the upper limit for the 90% confidential interval of RMSEA for Models A, B, and F is below the lower limit of RMSEA for Models C–E and G.

Although similar to Model F, Model H with an additional method factor did not converge to a solution with either correlated (*DF* = 164) or uncorrelated (*DF* = 165) method factors because the correlation matrix among the latent variables (phi matrix) was not positive definite. The same problem occurred for two versions of Model I. In the first version, the positive factors correlated with the other positive factors and the negative factors correlated with the other negative factors (*DF* = 164). In the second version, depicted in **Figure 1**, all six factors correlated with each other (*DF* = 155). The phi matrix was not positive definite in any version. Finally, several parameter matrices in Model J were not positive definite, and no solution could be found.

The fit of Model B, in which the *Autonomy* and *Competence* factors were collapsed, was as good as that of Model A. This indicates that *Autonomy* and *Competence* are hard to distinguish, something also seen by both the high correlation between the latent factors (*r* = 0.93–0.94) and the Modification Index (MI) of Models A and F that suggest cross-loadings between *Autonomy* and *Competence* for Q4, Q8, and Q13. Yet, the correlation between *Autonomy* and *Relatedness* was also substantial (*r* = 0.72–0.73) and cross-loadings are suggested here by the MI between *Autonomy* and *Relatedness* for Q1 and Q2. The correlation between *Competence* and *Relatedness* was 0.67, with the MI suggesting a cross-loading between *Competence* and *Relatedness* for Q21. The MI of Model F also suggested cross-loadings between Q1, Q2, and Q9 and the method factor.

Models A and F are interesting to compare in more detail because although they deal with the reversed items in different ways, the overall fit is about the same. The parameter values of each model are specified in **Table 3**. First, the loadings on *Autonomy*, *Competence*, and *Relatedness* were close to identical in the two models. All but the loadings on Q3 and Q19 were significant. The error variance for each of the nine reversed items in Model A was allowed to correlate with the other eight reversed items, and the sum of these correlations is shown in **Table 3** (Σ Correlated Error). In Model F, the reversed items were set to load onto a special method factor. The magnitude of these loadings was close to the magnitude of the correlated errors as shown in **Table 3**. All reversed items except Q19 loaded significantly onto the method factor. The uncorrelated errors were slightly higher in Model A compared with Model F for the reversed items, but identical for all others.

**Table 3:** Error variance and factor loadings for Model A and Model F (standardized values, CS, *N* = 600).

Indicator	Model A			Model F		
Need	Loading needs	Σ Correlated error	Uncorrelated error	Loading needs	Loading method	Uncorrelated error
*Autonomy*		
Q1^∗^	0.50	–	0.75	0.50	–	0.75
Q5 (R)	0.21	0.24	0.95	0.21	0.24	0.90
Q8^∗^	0.60	–	0.63	0.61	–	0.63
Q11 (R)	0.14	0.23	0.98	0.15	0.22	0.93
Q13^∗^	0.71	–	0.50	0.71	–	0.50
Q17	0.55	–	0.70	0.55	–	0.70
Q20^∗^ (R)	0.31	0.35	0.91	0.31	0.33	0.80
*Competence*		
Q3 (R)	0.01	0.26	1.00	0.00	0.26	0.92
Q4^∗^	0.67	–	0.54	0.68	–	0.54
Q10^∗^	0.41	–	0.83	0.41	–	0.83
Q12^∗^	0.52	–	0.73	0.52	–	0.73
Q14^∗^ (R)	0.42	0.36	0.83	0.42	0.34	0.70
Q19 (R)	0.12	0.42	0.99	0.11	0.42	0.81
*Relatedness*		
Q2^∗^	0.78	–	0.39	0.78	–	0.39
Q6	0.70	–	0.51	0.70	–	0.51
Q7 (R)	0.32	0.28	0.90	0.33	0.29	0.81
Q9	0.69	–	0.52	0.69	–	0.52
Q15^∗^	0.78	–	0.39	0.78	–	0.39
Q16^∗^ (R)	0.39	0.01	0.85	0.40	0.01	0.84
Q18 (R)	0.45	0.29	0.80	0.46	0.27	0.72
Q21^∗^	0.58	–	0.66	0.58	–	0.66

*All loadings except for Q3, Q19 are significant, *p* < 0.01; “(R)” indicates reversed items; asterisk “^∗^” denotes items included in the 12-item scale*.

Although mostly significant, it is striking how low many of the factor loadings are and how high the corresponding uncorrelated error variance is. This raises the issue of eliminating some indicators. The factor loadings from Models A and F were also used in developing the short scale. The loadings from Model B are of little interest, because this model is not supported by SDT.

### Scale Reduction

When reducing the scale, both psychometric and conceptual aspects were considered. We began by eliminating all indicators with a loading of below 0.30 (cf. Brown, 2015). Items Q3, Q5, Q11, and Q19 were eliminated on these grounds. This left three positive and one reversed item in the *Competence* factor, and so we decided to keep this mixture in the two remaining factors too. Thus, one more positive indicator needed to be eliminated from the *Autonomy* factor. Continuing to eliminate the item with lowest loading in *Autonomy* would have eliminated item 1, with a loading of 0.50 and error of 0.75. However, this item (*I feel like I can make a lot of input into deciding how my job gets done*) seems central to the concept of autonomy and therefore item 17 (*I feel like I can pretty much be myself at work*) with a loading of 0.55 and an error of 0.70 was eliminated instead. The factor loadings for *Relatedness* were generally good, although as with the other factors, lower for the negative items. The wording of Q18 (*The people I work with do not seem to like me much)* includes a demeaning component to which people are likely to react strongly regardless of how correct the statement is. Most respondents disapproved of this statement, resulting in it having the highest mean and kurtosis of all after reversal (*M* = 6.26 on a 7-point scale), and so this item was eliminated. In the choice between the two remaining negative items, we chose Q16, which loaded higher on the *Relatedness* factor and had a lower error variance than Q7. In addition, the wording is also somewhat sharper. Q6 is a very general statement that most people agreed with. It also had high kurtosis, and Q6 was therefore eliminated. Finally, Q9 shared a substantial part of the error variance with Q15, and because this was undesirable Q9 was eliminated. Q21 was chosen as the fourth item for measuring *Relatedness*. The items included in the reduced scale are marked in **Tables 1**, **3**. The factor loadings and error variances for the reduced scale changed only marginally from that of the full scale (between -0.03 and 0.03). The internal reliability (based on 1,200 participants) of the reduced scale was unchanged for *Autonomy* (0.63), slightly higher for *Competence* (0.60) but lower for *Relatedness* (0.63) (see also **Table 1**).

**Table 4** displays the fit indices for the 21-item and the 12-item scales for both CS and VerS. CS data from the reduced scale fitted Model A better than the data from the 21-item scale did, with higher CFI and TLI, lower SRMR and AIC, and equal RMSEA. The difference was more pronounced in the data from VerS, with all indices being superior for the 12-item scale (A chi-square comparison between the models was not possible because of the different number of indicators). The reduced scale also fitted Model B almost as well (χ^2^[CS] = 161.19, *DF* = 50, RMSEA = 0.061, SRMR = 0.045, CFI = 0.96, TLI = 0.95, and AIC = 217.19; χ^2^[VerS] = 163.79, *DF* = 50, RMSEA = 0.062, SRMR = 0.046, CFI = 0.97, TLI = 0.96, and AIC = 219.79). However, a chi-square test revealed a significant difference between Models A and B in VerS; Dχ^2^ = 6.62, D*DF* = 2, *p* = 0.045. Model F did not converge to an admissible solution for either sample. We therefore stopped further investigation of Models B and F.

**Table 4:** Test of measurement invariance of Basic Psychological Needs at Work Scale with 21 and 12 items in two random samples.

	χ^2^	*DF*	Δχ^2^	Δ*DF*	RMSEA (90% CI)	SRMR	CFI	TLI	AIC
21-Item scale		
CS (*n* = 600)	489.56	150			0.061 (0.055–0.068)	0.049	0.95	0.93	651.56
VerS (*n* = 600)	570.47	150			0.068 (0.063–0.074)	0.054	0.95	0.92	732.47
Invariance test		
Configural invariance	1060.03	300			0.066 (0.061–0.069)	0.054	0.95	0.92	1384.03
Weak invariance	1091.32	318	31.29^∗^	18	0.064 (0.060–0.068)	0.059	0.95	0.93	1379.32
Scalar invariance	1846.58	357	786.55^∗∗∗^	57	0.083 (0.080–0.087)	0.120	0.90	0.88	2140.58
Strict invariance	1977.84	420	917.81^∗∗∗^	120	0.079 (0.075–0.082)	0.120	0.90	0.90	2145.84
12-Item scale		
CS (*n* = 600)	156.19	48			0.061 (0.051–0.072)	0.044	0.96	0.95	216.19
VerS (*n* = 600)	157.57	48			0.062 (0.051–0.073)	0.045	0.97	0.96	217.57
Invariance test		
Configural invariance	313.76	96			0.062 (0.054–0.069)	0.045	0.97	0.95	433.76
Weak invariance	325.36	105	11.60	9	0.059 (0.052–0.067)	0.051	0.97	0.96	427.36
Scalar invariance	594.39	126	280.63^∗∗∗^	30	0.079 (0.072–0.085)	0.094	0.93	0.93	702.39
Partial scalar invar.	326.95	84	13.19	(-)12	0.069 (0.062–0.078)	0.080	0.95	0.95	418.95
Strict invariance	629.59	147	313.76^∗∗∗^	51	0.074 (0.068–0.080)	0.097	0.93	0.94	659.59

^∗^*p < 0.05, ^∗∗^. *p* < 0.01, ^∗∗∗^*p* < 0.001*.

### Invariance Testing

We continued to evaluate the Swedish version of the BPNS-W scale by investigating invariance across two random samples of Model A in both the long and the reduced version. The fit for Model A in the original 21-item version in CS and VerS is shown in the top two lines of **Table 4**. It can be noted that the fit was better for CS on most fit indices. Configural invariance was performed by multi-group CFA (MGCFA) to test whether the same factor structure fit both CS and VerS. Configural invariance constitutes the standard by which more constrained models are compared. Its chi-square and DF can be computed by simply adding the values from the two individual samples (given the same DFs in both) as well as in a MGCFA. The associated fit indices were reasonable. The following test is often referred to as either weak invariance, metric invariance or simply equal form, and is used to test fit when factor loadings are held constant across groups. The same three indicators (1Q for *Autonomy*, 2Q for *Relatedness*, and 12Q for *Competence*) served as references for scaling the loadings when testing the equality of factor loadings for the long and short models. Although the fit indices were still reasonable, there was a significant increase in the chi-square (Δχ^2^ = 31.29, *DF* = 18, *p* = 0.027). The next test was crucial and concerns the latent means of the three needs held constant across the two samples. It is called scalar invariance and determines whether it is legitimate to use statistics on manifest variables based on mean comparisons. The fit indices for scalar invariance were poor, and the increase in chi-square huge (Δχ^2^ = 786.55, *DF* = 57, *p* < 0.001). Finally, strict invariance, in which the error variance for each pair of indicators in the two groups is also held equal, was not supported. However, strict invariance is seldom achieved and rarely needed (see **Table 4**).

Turning to invariance testing of the reduced scale, we noted first that the fit for CS and VerS was good and quite similar (**Table 4**). The configural test fitted the data well, as did the test of weak invariance. The change in chi-square between the two models was not significant. The third test was for scalar invariance and tested whether the latent means of *Autonomy*, *Competence*, and *Relatedness* were different in the two groups. This was found to be the case; this model was inferior in fit to the configural model, as revealed by all fit indices and confirmed by a significant increase in chi-square. However, the fit was still adequate according to some of the more liberal criteria for model fit (e.g., Browne and Cudeck, 1993). A closer inspection revealed that the difference in latent means was highest for *Relatedness*, with a difference of 0.09 between the two groups compared with a difference of 0.03 for *Autonomy* and 0.05 for *Competence*. We therefore relaxed the constraints for scalar invariance to achieve a partial scalar invariance, as discussed by Byrne et al. (1989). It was found that Q14 and Q16 were responsible for much of the variation in means between CS and VerS. These two indicators were therefore excluded from the partial model. In addition, the error variance between Q13 and Q15 was set free to vary. After these modifications, the model fit was no longer different from that of the configural model and partial scalar invariance between CS and VerS was hence supported. As with the full scale, strict invariance, in which the error variance for each pair of indicators in the two groups is also equal, was not supported.

### Validation

We used only the reduced scale in this analysis because the long scale did not quite pass the test for partial scalar invariance and hence was not suitable for the regression analysis we had planned. The correlation coefficients between the included variables are shown in **Table 5**. Although all correlations were significant, their magnitude did not rule out a regression analysis. A hierarchical regression with psychological well-being as dependent variable and *Autonomy*, *Competence*, and *Relatedness* entered as independent variables in that order in separate blocks revealed that each need made a unique contribution to psychological well-being. *Autonomy* alone explained 24.4% of the variance in psychological well-being, *Competence* accounted for an additional 9.2% of the variance and *Relatedness* an additional 2.0%. The contribution of each step was significant, *F*(1,415) = 13.211, *p* < 0.001 for the last step. Together, the model explained 35.3% (adjusted) of the variance. With a tolerance over 0.50 and VIF below 2.00, multicollinearity was not an issue. See **Table 6** for the regression coefficients after all three steps and collinearity statistics.

**Table 5:** Pearson correlations between psychological well-being (GHQ) and a composite score of each basic need on the reduced scale (manifest values).

	GHQ	*Autonomy*	*Competence*	*Relatedness*
GHQ	1			
*Autonomy*	0.494	1		
*Competence*	0.544	0.616	1	
*Relatedness*	0.377	0.390	0.384	1

*N = 419. All correlations are significant at the 0.01 level*.

**Table 6:** Regression coefficients and collinearity statistics when *Autonomy*, *Competence*, and *Relatedness* from the reduced scale are regressed against psychological well-being (GHQ).

	*B*	SE *B*	b	*t*	Tolerance	VIF
*Autonomy*	0.089	0.021	0.217	4.253	0.593	1.686
*Competence*	0.145	0.021	0.349	6.853	0.596	1.677
*Relatedness*	0.073	0.020	0.158	3.635	0.815	1.228

*All *p* < 0.001*.

## Discussion

This study found support for a three-factor structure of a Swedish version of the original BPNS-W scale comprising 21 items and correlated error variance between reversed items. A reduction to 12 items improved most psychometric properties, and partial invariance between CS and ValS was claimed for the short but not the long version. Finally, data from the 12-item scale related significantly to psychological well-being, and each need made a significant contribution to the explained variance in well-being. The latter result constitutes independent evidence for a three-factor structure.

Three-factor structures fitted the data from the Swedish adaptation of the original 21-item version of BPNS-W satisfactorily and equally well. One (Model A) was the three-factor model supported by SDT (Deci et al., 2001) and for which related scales have been supported (Vlachopoulos and Michailidou, 2006; van den Broeck et al., 2010; Ng et al., 2011; Brien et al., 2012). However, the success of Model A was dependent on allowing the error variances to correlate for the reversed items. This feature is very similar to including a separate method factor for the negatively worded items, as was done by Johnston and Finney (2010) and depicted in Model F. Which method is the theoretically more satisfying is debatable (for a discussion on this, see Horan et al., 2003; DiStefano and Motl, 2006; Maul, 2013). Yet, the higher robustness of models with correlated errors for the reversed items compared with that achieved by adding a method factor (cf. Marsh and Grayson, 1995) became decisive in this study as Model F did not converge for the reduced scale.

Model B was also supported by the 21-item scale. This indicates that correlation between the factors of *Autonomy* and *Competence* was high in Model A. Consequently, the MI indicated several cross-loadings between *Autonomy* and *Competence*, although there were other cross-loadings as well. Hence, the success of model B points to a serious weakness of Model A, a weakness that carried over to the 12-item version (*r*[AC] for the 21-item version was 0.94 and 0.90 in the two samples, compared with 0.94 and 0.92 for the 12-item version). Model E, with only one latent factor, was mentioned as a viable model by Deci et al. (2001) in addition to Model A. It is therefore important to point out that Model E received no support in this study, corroborating the results of Vlachopoulos and Michailidou (2006).

Models H (Sheldon and Hilpert, 2012), I (Chen et al., 2015), and J (Longo et al., 2016) did not converge to permissible solutions. Small sample size is a common cause of inadmissible solutions. However, the present sample contained 600 participants, which should have been enough. One reason for the failure to converge is probably that the models were primarily developed for scales concerned with the inclusion of a frustration dimension. Yet, Sheldon and Hilpert (2012) claimed that Model H also fitted the original 21-item scale (general version) well, although they additionally reported that some extra constraints needed to be imposed for the model to converge. The negatively worded scales in the original BPNS are more heterogeneous than these specifically designed frustration dimensions and some of their error variance might be negative, something that can disturb the fit of most models.

The need to include negatively worded items could also be questioned. As pointed out by DeVellis (2012) and demonstrated in this study, negatively worded items often cause problems in factor structures, here in the form of correlated error variance or designated method factors. It has also been argued that participants are often confused when asked to agree with negatively worded items (e.g., De Vaus, 2013). The arguments for including negatively worded items are to induce more variation in the questionnaire and to guard to some extent against acquiescence bias (Spector, 1992). However, these are surface features of the instrument and having negatively worded items in the questionnaire does not necessarily mean they have to be included in the analyses.

The reduced, 12-item three-factor model with correlated error variance between the three reversed items resulted in a slightly better fit of the data compared with the 21-item version, not only in CS but more importantly also in VerS. Partial scalar invariance was also achieved for the short model. It may be argued that partial scalar invariance could also be achieved for the long version; that it is just a matter of how much you modify the original model. Byrne et al. (1989) asserts a minimum of two unmodified items per factor as a guideline. However, we believe a reduced scale with few modifications is to be preferred to a longer scale with more modification to reach partial scalar invariance. Yet, the internal reliability of both the long and short scales was poor. It might therefore be fruitful to explore whether there are more dimensions than the three previously considered. A close inspection of the items designated to measure *Relatedness*, for example, reveals that most items are about a person’s attitudes toward the people he/she works with (Q2, Q6, Q7, Q9, and Q16) whereas the other items concern the attitudes of the people the person works with toward that person. Similarly, *Competence* is both about how well the person masters the skills of her job and the respect others pay that person for those skills. It is less clear whether *Autonomy* includes more than one dimension. These issues could be refined in future studies on how *Autonomy*, *Competence*, and *Relatedness* are best measured, including the formulation of new items and how they work in different language societies.

## Conclusion

In conclusion, we find that the 12-item version of the Swedish adaptation of the BPNS-W has some limitations but also some considerable strengths, including a three-factor structure, as predicted by theory, partial scalar invariance, and external validation using psychological well-being as criterion. Further development of the scale is desirable, however, in particular with the aim of increasing its internal consistency, relaxing the correlation between *Autonomy* and *Competence* and reducing the high error variance associated with some items. Further comparisons between the long and the short versions of the scale in non-Swedish languages are also needed. Further validation of the scale would also be welcome, in particular in relation to intrinsic work motivation. Meanwhile, we recommend the use of the 12-item version presented here over that of the original 21-item version in studies where psychological needs at work are related to psychological well-being or related constructs in an SDT framework.

## Author Contributions

EB and ME generated jointly the idea to the study and both supervised the data collection. ME analyzed the data and drafted the manuscript. EB provided critical comments and suggestions continuously resulting among other things in an improved structure. Both authors gave consent to the final version and take responsibility for the work.

## Conflict of Interest Statement

The authors declare that the research was conducted in the absence of any commercial or financial relationships that could be construed as a potential conflict of interest.

## Footnotes

1 http://www.selfdeterminationtheory.org
